# The Role of Ascorbic Acid Added to Wine in the Corrosion Process of Stainless Steel Used in the Wine Industry

**DOI:** 10.3390/ma19091872

**Published:** 2026-05-01

**Authors:** Mircea Laurențiu Dan, Nataliia Rudenko, George-Daniel Dima

**Affiliations:** 1 Laboratory of Electrochemistry, Corrosion and Electrochemical Engineering, Faculty of Chemical Engineering, Biotechnologies and Environmental Protection, University Politehnica Timisoara, 6 Pârvan, 300223 Timisoara, Romania; mircea.dan@upt.ro; 2 Innovation and Technology Transfer Center, University Politehnica Timisoara, 307221 Timisoara, Romania; nataliia.rudenko@upt.ro

**Keywords:** ascorbic acid, stainless steel 304, wine industry, corrosion inhibition, corrosion techniques, adsorption isotherms, electrochemical impedance spectroscopy

## Abstract

This paper presents the electrochemical behavior of stainless steel 304 (SS304), a material often utilized in the wine industry, in the presence of varying concentrations of ascorbic acid (AcAS), introduced in a neutral solution (Na_2_SO_4_ 0.25 M + 12% (*v*/*v*) EtOH). The experimental part of this paper included potentiodynamic polarization and chronoamperometry techniques to evaluate the influence of ascorbic acid on the corrosion processes in the test solutions. Electrochemical impedance spectroscopy (EIS) has been used to investigate the charge transfer at the interface and the formation of a protective film in the absence and presence of AcAS. The Tafel method was employed to determine the kinetic parameters of the corrosion process studied. Additionally, several models of adsorption isotherms were applied to describe the interactions between AcAS and the stainless steel surface, with the Freundlich and Dubinin–Radushkevich isotherms demonstrating the most robust correlation, based on the R^2^ correlation coefficients. Quantum chemical calculations (DFT) were also performed to clarify the molecular mechanism via which AcAS functions as an eco-friendly corrosion inhibitor in winemaking-related environments.

## 1. Introduction

Corrosion is an inherent natural process that impacts all metallic materials subjected to aggressive environments [1]. This process not only leads to substantial economic losses due to maintenance, repair, and replacement of corroded structures but also raises significant safety and environmental issues [2]. Industries including construction [3], energy production [4], transportation, and marine engineering experience significant corrosion-related damage, highlighting the necessity for effective and sustainable protection methods [5].

Traditional strategies for corrosion prevention—such as surface coatings, cathodic protection, and the addition of corrosion inhibitors—have demonstrated efficacy, although frequently entail disadvantages [6]. Numerous traditional corrosion inhibitors are toxic, non-biodegradable, and may endanger human health and the environment. In recent decades, heightened awareness of environmental sustainability and stricter environmental regulations have stimulated an urgent quest for eco-friendly, biodegradable, and non-toxic corrosion inhibitors, also known as green inhibitors [7,8].

In parallel with the growing demand for safer additives in industrial technologies, L-ascorbic acid, or AcAS, is widely used as an antioxidant in the alcoholic beverage industry, particularly in winemaking, where it acts as a “sacrificial” substrate for dissolved oxygen and derived radicals, thereby limiting the oxidation of phenolic compounds and the volatile aromatic fraction. L-ascorbic acid plays a dual and concentration-dependent role in wine oxidation, acting both as a protective antioxidant and, under certain conditions, as a pro-oxidant species [9]. Barril et al. showed that ascorbic acid in white wine can effectively scavenge dissolved oxygen and delay the development of oxidative aroma defects, but also highlighted that its degradation leads to the formation of hydrogen peroxide and other reactive intermediates that may accelerate phenolic oxidation if not adequately controlled by sulfur dioxide [10]. Other reports further emphasized the critical role of ascorbic acid in modulating the redox balance of wine, demonstrating in model systems that the ascorbic acid/dehydroascorbic acid couple participates in complex reaction networks with phenolics, metals, and oxygen, ultimately influencing browning susceptibility and color stability. Consistent with these findings, Gibson et al. described ascorbic acid as both “friend and foe” in winemaking, underlining that its technological benefits in preventing pinking and preserving fruity aromas are maximized only when oxygen exposure, metal content, and free SO_2_ levels are tightly managed [11]. Overall, these studies converge on the view that ascorbic acid is not a simple antioxidant additive, but a reactive redox mediator whose impact on wine oxidation strongly depends on matrix composition, storage conditions, and its use in combination with sulfur dioxide and other antioxidants [9,10]. From a regulatory standpoint, ascorbic acid is approved as a food additive in the European Union for wine, aromatized wines, and other fermented beverages, with specific maximum permitted levels (on the order of 25 g/hL for grape wine) established to ensure both consumer safety and technological efficacy in products such as wine, cider, and other fermented beverages [9,10,11].

Among the various organic compounds explored for this purpose, ascorbic acid (vitamin C) has attracted considerable attention due to its unique molecular characteristics and biological origin. As a naturally occurring water-soluble vitamin, ascorbic acid is essential for human metabolism and possesses well-known antioxidant properties [12]. Chemically, it possesses hydroxyl (-OH) and carbonyl (C=O) functional groups that can donate electron pairs, promoting adsorption onto metal surfaces and creating a protective barrier against corrosive agents. Its rapid availability, affordability, and non-toxic characteristics make it an excellent alternative to conventional, hazardous inhibitors in industrial applications [13].

The efficacy of ascorbic acid as a corrosion inhibitor was initially emphasized by Sekine, Nakahata, and Tanabe, who examined its influence on mild steel corrosion in sodium chloride solutions. Their findings indicated that ascorbic and folic acids may markedly reduce corrosion rates, with inhibition attributed to chemical adsorption in accordance with the Langmuir isotherm model. Significantly, they noted that inhibition efficacy decreased at excessively high concentrations due to the formation of iron–ascorbate complexes that accelerated corrosion at those levels [14].

Subsequent decades saw subsequent investigations that expanded this basis into more industrially relevant systems. Fuchs-Godec and Pavlović performed comprehensive electrochemical investigations on stainless steel (SS X4Cr13) immersed in hydrochloric acid, a medium characteristic of pickling and cleaning processes. Utilizing potentiodynamic polarization and impedance spectroscopy, they established that ascorbic acid functions as a mixed-type inhibitor, concurrently reducing both anodic dissolution and cathodic hydrogen evolution. The formation of iron–ascorbate chelates on the steel surface was supported by energy-dispersive X-ray analysis, confirming the adsorption mechanism and its protective role [15,16].

Simultaneously, research conducted by Anejjar et al. investigated ascorbic acid’s efficacy in carbon steel corrosion inhibition under acidic media (1 M HCl), utilizing weight-loss, polarization, and impedance measurements. Their findings corroborated earlier studies, demonstrating that inhibition efficiency increased with inhibitor concentration and that adsorption followed the Langmuir isotherm. Thermodynamic parameters indicated spontaneous adsorption, emphasizing a predominantly chemical interaction between ascorbic acid molecules and the metal surface [17].

Beyond acidic environments, researchers have investigated the inhibitor’s efficacy in more intricate and realistic systems. Kasatkin et al. studied ascorbic acid in model concrete pore solutions with chlorides and determined that it effectively protected steel reinforcement by mitigating chloride-induced corrosion. The optimum concentration for long-term stability was determined to be around 1 g/L, above which efficiency declined due to excessive chelation effects [18].

Similarly, Irwan et al. evaluated the corrosion of low-carbon steel in simulated seawater (3.5% NaCl), revealing that inhibition efficiency exceeded over 85% after 15 days of immersion at an ascorbic acid concentration of 50 ppm. These findings robustly indicate the applicability of vitamin C in marine and coastal environments [19].

Experimental observations have confirmed the effectiveness of ascorbic acid as a green corrosion inhibitor, while subsequent computational studies have elucidated molecular interactions with metallic surfaces. Employing density functional theory (DFT) and tight-binding methodologies, Souza et al. compared the adsorption behaviors of ascorbic acid, ascorbate, and dehydroascorbic acid on the α-Fe (110) surface. Simulations indicated that dehydroascorbic acid demonstrated the highest adsorption energy and charge-transfer interaction, indicating superior protective potential. Theoretical insights enhance experimental data and enrich our comprehension of the electronic structure–activity relationships that dictate corrosion inhibition [20].

Furthermore, the safety data sheets (SDS) and technical parameters of ascorbic acid further substantiate its environmental compatibility. In contrast to the majority of synthetic inhibitors that contain heavy metals or phosphates, ascorbic acid is non-toxic, non-carcinogenic, and entirely biodegradable. Industrial-grade products comply with European regulatory standards (EU 2019/934) and are extensively utilized as antioxidants and preservatives in the food and beverage sector [21,22].

Overall, these aspects justify the evaluation of AcAS as a food-compatible corrosion inhibitor for SS304 stainless steel under conditions similar to beverage industry environments. This study will investigate the corrosion behavior of SS304 in 0.25 mol L^−1^ Na_2_SO_4_ with 12% EtOH (*v*/*v*) in the presence and absence of different concentrations of AcAS, in the temperature range of 298–328 K, using cyclic voltammetry, linear voltammetry, chronoamperometry, chronopotentiometry and electrochemical impedance spectroscopy as techniques; integrated with DFT methods and adsorption isotherms. The integrated approach correlates electrochemical performance, temperature influence and interactions at the molecular level, clarifying how AcAS behaves electrochemically at the surface level of the studied steel, under conditions relevant to the wine industry.

## 2. Materials and Methods

### 2.1. Materials

**Figure 1:**
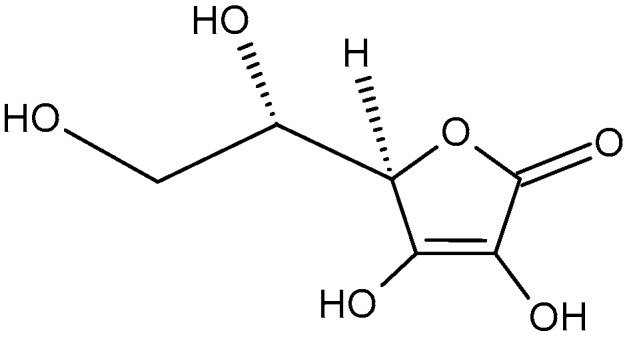
Structure of L-ascorbic acid.

The molecular formula of AcAS is C_6_H_8_O_6_ and the name according to IUPAC nomenclature rules is (4*R*,5*R*)-5-[(1*S*)-1,2-dihydroxyethyl]-4-hydroxyoxolane-2,3-dione [23]. From the point of view of the class of compounds to which it belongs, it is an enediolic gamma-lactone and is synthesized by several plants, such as citrus fruits: lemon, orange, rosehips, peppers, kiwis, and sea buckthorn [24]. Industrially, AcAS is synthetically obtained through the Reichstein process, modern processes employing two-step microbial fermentation [25]. It is commercially available as tablets for consumption, and as a reagent, it is found in the optical L form, produced by Sigma-Aldrich (St. Louis, MO, USA), with a concentration of 99%. Figure 1 illustrates the structure of ascorbic acid.

AcAS shows high stability in the acidic environment, and in neutral and especially alkaline environments, it undergoes oxidation to dehydroascorbic acid, followed by degradation [26].

The electrochemical determinations conducted to analyze the electrochemical behavior of AcAS on the corrosion of the studied material, used in the food industry, were performed using the BioLogic SP150 potentiostat—galvanostat (BioLogic Science Instruments, Seyssinet-Pariset, France), connected to a non-compartmentalized electrochemical cell equipped with three electrodes, a working electrode made of SS304 steel (with an active surface of 0.785 cm^2^), two cylindrical counterelectrodes made of graphite (having a submersible size of 5 cm and a base diameter of 0.7 cm), and an Ag/AgCl reference electrode, at a potential of 0.197 V vs. SHE, for measuring the potential of the working electrode. All potential values presented below are referenced to the value of the reference electrode. Table 1 illustrates the elemental composition of SS304 steel used in experimental determinations. In particular, the contents of Cr and Ni are reported as permissible specification ranges, and Fe represents the balance.

The electrolyte support (BS) solution consisted of Na_2_SO_4_ 0.25 mol L^−1^, with the addition of 12% ethyl alcohol. As this is an important study for the wine industry, a concentration of 12% (*v*/*v*) EtOH was chosen, similar to that existing in these alcoholic beverages. Before each electrochemical test, through the experimental cell containing 100 mL of electrolyte, nitrogen—N_2_—was purged for 10 min at a constant flow rate of 100 mL/min to remove the dissolved oxygen. To investigate the effect of concentration on corrosion rate and inhibitory efficacy of *IE*, tests were performed at working concentrations of 12.5; 25; 50; 75; 100; and 150 mg L^−1^ AcAS, added to the mentioned BS electrolyte support solution. The electrolyte solutions used had pH values ranging from 4.47 to 5.57 (±0.1), corresponding to concentrations from 150 mg L^−1^ to 12.5 mg L^−1^. The reagents utilized were: L-ascorbic acid, 99% powder; Na_2_SO_4_ ACS reagent, >99%; and Ethanol ≥ 95.0%, ACS spectrophotometric grade, all produced by Sigma-Aldrich (St. Louis, MO, USA).

### 2.2. Electrochemical Methods

The electrochemical measurements were conducted with the BioLogic SP150 potentiostat (BioLogic Science Instruments, Seyssinet-Pariset, France), controlled by the EC-Lab v. 10.23 software (BioLogic Science Instruments, Seyssinet-Pariset, France). Cyclic voltammetry tests utilized platinum as the working electrode, conducted at polarization rates of 10 and 100 mV s^−1^, within potential ranges of −0.75 to 2.5 V. The electrochemical techniques for the study of the corrosion process, specifically linear voltammetry, chronopotentiometry, chronoamperometry and electrochemical impedance spectroscopy, were conducted on the SS304 working electrode three times to ensure reproducibility of the experiment. Before starting each experimental technique, the surface of the working electrode was cleaned by sanding with Si-C abrasive papers of 80, 120, 240, 600, 1200 and 2400 grit, followed by ultrasonication, alcohol cleaning and drying. From the linear voltammograms plotted at the rate of 1 mV s^−1^ in the potential range + 0.5 V and −0.1 V relative to the *E*_OCP_, by applying logarithmic transformation, the parameters of the Tafel slopes, specifically the corrosion current *i*_corr_, anodic slope *b*_a_, cathodic slope *b*_c_ and *E*_corr_ are derived. These parameters aid in the calculation of the corrosion rate *v*_corr_ and inhibitory efficiency *IE*, as described in Equation (1) [27].

(1) $$IE=\frac{i_{corr,a}-i_{corr,p}}{i_{corr,a}}\cdot 100=\theta \cdot 100$$

where

*IE*—inhibitory efficacy [%];

θ—surface coverage degree;

$i_{corr,a}$ and $i_{corr,p}$—the corrosion current for the process carried out in the absence and the presence of AcAS [A m^−2^], respectively.

Electrochemical impedance spectroscopy (EIS) was utilized to investigate the mechanism of electrochemical behavior of SS304 steel in BS in the presence and absence of different concentrations of AcAS. All spectra were plotted at the potential value corresponding to *E*_OCP_, on a frequency range between 10^6^ and 10^−2^ Hz, at the value of the oscillation amplitude of 10 mV, on 60 points with a logarithmic distribution of 10 points per decade. The method of the smallest squares (Levenberg–Marquardt) was used, and the 60 points were fitted into an EEC equivalent electrical circuit utilizing the ZView program, version 4.0, Scribner Associates Inc., Southern Pines, NC, USA [28].

### 2.3. Molecular Modeling

In order to provide a supplement to the results from voltametric tests, theoretical calculations of molecular modeling were performed utilizing the Hyperchem program, v. 8.0. The AcAS molecule was simulated in aqueous and vacuum conditions using DFT with the B3LYP function and the 6-31G* basis set. Subsequent to the optimization of the studied molecule, the energies of the Frontier orbitals were obtained: the higher occupied molecular orbital—E_HOMO_, the lower unoccupied molecular orbital—E_LUMO_, and the associated descriptors derived from them, such as HOMO-LUMO energy gap (∆*E*), chemical hardness (*η*), chemical softness (*σ*) and dipole moment (*μ*). Structurally, a larger molecule can prevent further corrosive attacks on the metal surface. Properties such as molecular volume, *V*, molecular surface *S*, and their ratio were obtained using the QSAR properties module of the employed software.

## 3. Results

### 3.1. Cyclic Voltammetry

**Figure 2:**
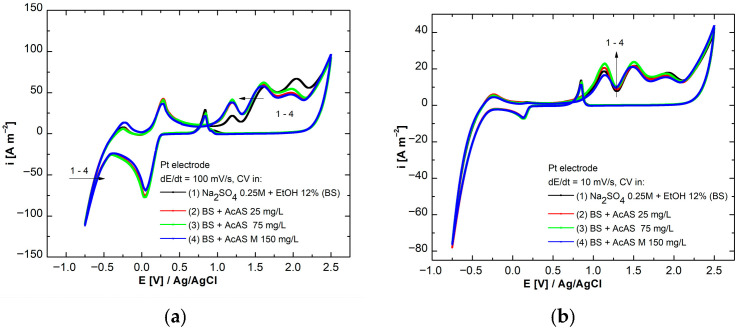
Cyclic voltammograms plotted on platinum electrode at different concentrations of AcAS at scan rates of (**a**) 100 mV s^−1^ and (**b**) 10 mV s^−1^.

In order to study the reaction mechanism at the level of the metal-aggressive solution interface, both in the presence and absence of AcAS, across the potential ranges between the hydrogen and oxygen evolution potential, cyclic voltammograms were obtained in BS—Na_2_SO_4_ 0.25 mol L^−1^ + 12% EtOH (*v*/*v*)—to which concentrations of 25, 75 and 150 mg/L AcAS were added. All cyclic voltammetry measurements were carried out at 298 K under thermostatic conditions. The determinations were performed to analyze the behavior of the studied compound on a platinum electrode with an active surface area of 0.5 cm^2^, at the polarization rates of 100 mV s^−1^ and 10 mV s^−1^, starting from OCP in anodic direction. The choice of the Pt electrode is justified by the fact that it provides a reproducible surface that allows the evaluation of the oxidation or reduction processes of AcAS between the hydrogen evolution potential and the oxygen evolution potential with minimal interference. Figure 2 illustrates the cyclic voltammograms plotted under the mentioned conditions.

In Figure 2a, at the scanning rate of 100 mV s^−1^, we observe a sequence of processes characteristic of redox reactions at the platinum surface. As the concentration of AcAS rises, a gradual decrease in the current density inside the positive potential frame (>+1 V compared to *E*_Ag/AgCl_) can be observed, which indicates that the studied molecule undergoes a stepwise oxidation process. At the level of the oxygen release process > 2.25 V, AcAS does not show an influence in this regard, the peaks remaining overlaid. The cathodic branch, starting with the potential E < 0.5 V/Ag/AgCl, highlights the peak reduction of the oxides formed [29]. Parallel to this, the reverse process of formation of superficial oxides on the surface of the platinum can be observed. Similarly, during the hydrogen release process, starting with a potential of less than −0.5, AcAS does not show any influence on it by increasing the concentration [30]. At a lower scanning rate (10 mV s^−1^; Figure 2b), we observe a more clearly defined structure of the anodic redox peaks associated with the oxidation of AcAS, the current density corresponding to those processes increasing with the addition of AcAS, its influence on the processes of gas release from the electrochemical decomposition of water being thus minimal.

**Figure 3:**
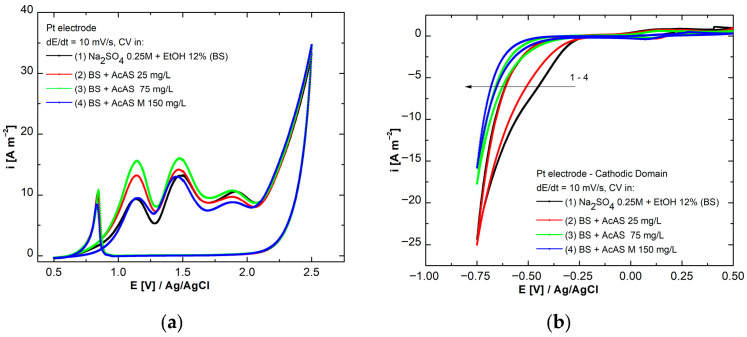
Anodic (**a**) and cathodic (**b**) domains for AcAS behavior on Pt electrode.

To facilitate a more detailed observation of the processes, separate cyclic voltammograms were plotted for both the anodic (Figure 3a) and the cathodic domain (Figure 3b) at the scan rate of 10 mV s^−1^.

Figure 3a is represented by the anodic domain and depicts a sequence of prominent peaks that are located between +1.0 and 2.2 V relative to *E*_Ag/AgCl_. Their intensities fluctuate according to the concentration of AcAS. The direct oxidation of the organic molecule on the Pt surface is attributed to them. Peak intensification when the concentration increases up to 75 mg/L suggests an active oxidation process, while at 150 mg/L, we observe a slight decrease in current due to a more pronounced surface saturation or inhibitory adsorption. Therefore, AcAS behaves as an electrochemical compound active in the anodic domain, with a progressive inhibitory effect on the overall oxidation process [29,30]. In the cathodic branch, Figure 3b highlights the influence of AcAS on the hydrogen release process. In comparison to BS, the curves associated with the concentrated increase exhibit a leftward shift in the hydrogen release potential towards more negative values, indicating an inhibition of the cathodic reaction and a postponement of the reduction process due to the adsorption of AcAS on the electrode surface, which is evidenced by the diminished accessibility of reactive species to the active surface [31].

### 3.2. Linear Sweep Voltammetry

**Figure 4:**
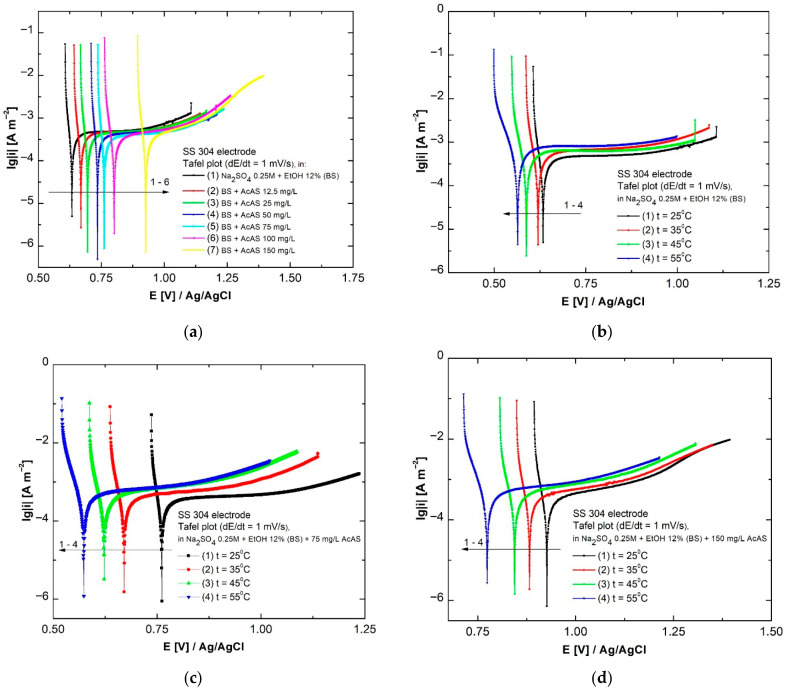
Potentiodynamic polarization curves for the SS304 electrode in BS and in the presence of AcAS (**a**); temperature influence at concentration 0 AcAS (**b**), 75 mg/L AcAS (**c**), 150 mg/L AcAS (**d**).

The impact of concentration on anode and cathodic slope alloying, together with corrosion currents and rates, was examined using linear voltammograms. These consisted of applying a linear variation in the potential over time with a scan rate of 1 mV s^−1^ within the potential range of +0.5 V/E_OCP_ and −0.1 V/E_OCP_ during the measurements. The linear voltammetry was conducted on the SS304 working electrode, and from the logarithmic form, the Tafel parameters were extracted, which provide information on the kinetics of the anodic and cathodic reactions involved in corrosion. Figure 4 represents the potentiodynamic polarization diagrams resulting from linear voltammetry in four experimental scenarios as follows: the influence of AcAS concentration at 25 °C (Figure 4a), the influence of temperature in the absence of AcAS (Figure 4b), the influence of temperature in the presence of 75 mg L^−1^ AcAS (Figure 4c), the influence of temperature in the presence of 150 mg L^−1^ AcAS (Figure 4d).

The results of the kinetic parameters derived from the Tafel slopes are illustrated in Table 1.

At 25 °C, the addition of varying concentrations of AcAS results in a significant decrease in the corrosion current density from 14.8·10^−3^ μA cm^−2^ in BS to 5.6·10^−3^ μA cm^−2^ with the addition of 150 mg L^−1^ AcAS. This trend is accompanied by a shift in *E*_corr_ towards more positive values, starting from 631 mV to 926.3 mV vs *E*_Ag/AgCl_, which indicates a higher thermodynamic stability of the surface. It should be noted that SS304 is a passive alloy, and in the low-aggressiveness electrolyte used in the study, Tafel extrapolation results in low current densities. Under these conditions, this method provides apparent values of *i*_corr_, with higher uncertainty on the anodic branch affected by passivation. Therefore, *i*_corr_ and *v*_corr_ are used in principle for comparative purposes rather than as absolute corrosion-rate values. From the decrease in both the anodic and cathodic slopes, a reduction in the rate of redox reactions at the interface is indicated, which correlates with the low values of *v*_corr_, from 0.158·10^−3^ to 43.01·10^−6^ mm y^−1^. This observation suggests an inhibitory effect of AcAS, especially at concentrations exceeding 75 mg/L, where the adsorption of the organic compound becomes efficient and stable. In the absence of AcAS (Figure 4b, Table 2), increasing the temperature from 25 °C to 55 °C considerably accelerates the corrosion processes. Specifically, each 10 °C increment raises the current density from 14.8·10^−3^ μA cm^−2^ to 26·10^−3^ μA cm^−2^, while the *v*_corr_ increases from 0.158·10^−3^ mm y^−1^ to 0.269·10^−3^ mm y^−1^. This behavior is also associated with a shift in the *E*_corr_ towards more negative values, which indicates an increased instability of the passive layer at the SS304 surface level. Tafel slopes exhibit an increase with temperature, suggesting a thermal activation of oxidation and reduction processes in the absence of effective inhibitory protection. In the case of the solution containing 75 mg L^−1^ AcAS (Figure 4c), the increase in quenching shows a more moderate impact on the corrosion process: *E*_corr_ decreases from 759.4 mV to 572.2 mV as the temperature rises, which is less pronounced than in the case of BS. In this case, the concentration of AcAS confers a significant degree of protection by physicochemical adsorption, efficient up to intermediate temperatures of 45 °C, but weakens at 55 °C. In the last case of the tested concentration, 150 mg L^−1^ AcAS (Figure 4d), the tested organic substance maintains its inhibitory effect even at high temperatures. Although the *i*_corr_ increase from 5.6·10^−3^ to 14·10^−3^ μA cm^−2^ occurs concomitantly with the *E*_corr_ shift from 926.3 mV to 774.1 mV, *v*_corr_ remains below the values obtained in the absence of AcAS, even at the final examined temperature 55 °C. This suggests the stability of the passive film through multi-hydrogen bonds and chelation between the functional groups of AcAS and the SS304 surface [32]. In comparison to the concentration of 75 mg L^−1^, a concentration of 150 mg L^−1^ AcAS provides enhanced and prolonged protection, especially under more extreme heat conditions.

### 3.3. Chronoamperometric and Chronopotentiometric Studies

**Figure 5:**
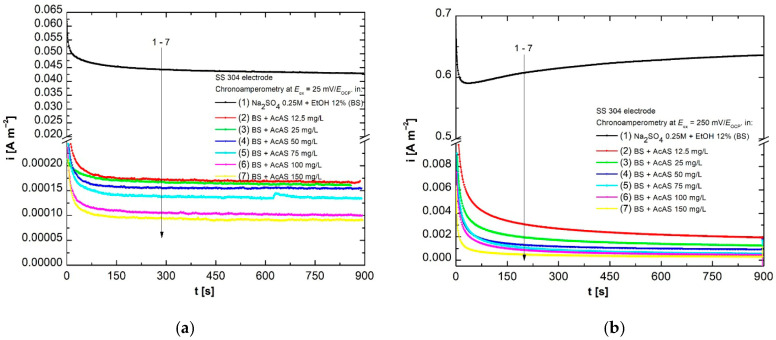
Chronoamperograms of SS304 in BS and with ACAS at +25 mV/*E*_OCP_ (**a**) and +250 mV/*E*_OCP_ (**b**).

From a principled point of view, the chronoamperometric method is based on recording the variation in the current density as a function of time, applying a well-defined potential to the working electrode. The above-mentioned technique was applied in order to highlight the influence of different concentrations of AcAS on the anodic oxidation process of SS304, in an environment of Na_2_SO_4_ 0.25 mol L^−1^ with 12% EtOH (*v*/*v*) (BS), to which the working concentrations of AcAS were added. For this study, determinations were conducted at fixed oxidation potentials of +25 mV/*E*_OCP_ (Figure 5a) and +250 mV/*E*_OCP_ (Figure 5b), for a duration of 900 s, at 298 K.

The analysis of Figure 5 shows that in both scenarios, the values of the oxidation current densities decrease significantly with the addition of different increasing concentrations of AcAS, which suggests the efficient inhibition of the oxidation process. A considerable decrease is observed at the last concentration, 150 mg L^−1^ AcAS, which indicates the formation of an efficient passive layer on the surface of the working electrode, reducing the corrosion rate. In the case of the potential of +250 mV/*E*_OCP_, the current values are higher compared to the case recorded at 25 mV/*E*_OCP_, and the tendency to reduce the i_ox_ current density is more evident, confirming the protective role of AcAS, which limits charge transfer through adsorption at the level of the metal surface.

**Figure 6:**
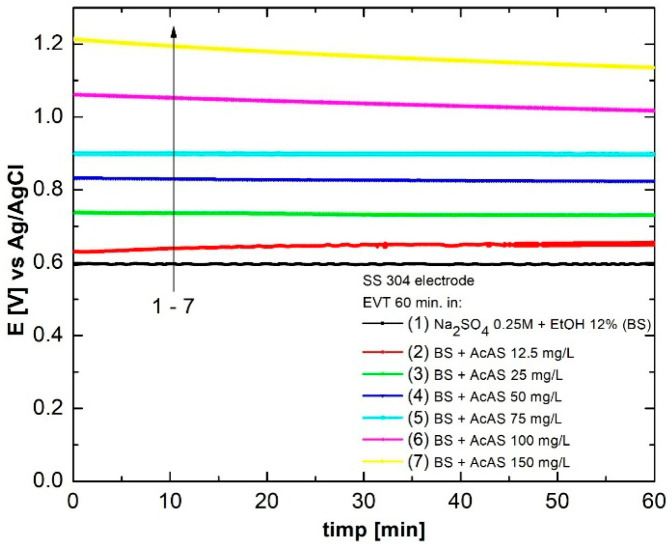
Evolution of open circuit potential for test solutions.

Before performing each corrosion determination, chronopotentiometry was used by monitoring the time of *E*_OCP_ evolution in the SS304 electrode under quasi-stationary conditions for 60 min, at varying concentrations of AcAS. Figure 6 represents the variation in the open circuit potential for the SB–SS304 system, but also in the presence of the six concentrations of AcAS: 12.5, 25, 50, 75, 100 and 150 mg/L. We observe that in all cases, as the AcAS concentration increases, the *E*_OCP_ value progressively increases, which indicates a progressive passivation of the metal surface, suggesting effective coverage of the active surface with inhibitor molecules.

In comparison to BS, the potential in the presence of AcAS shows more positive values, and the diagrams reach a quasi-stationary state about 20–30 min after exposure. The value of the stable potential is an indicator of the corrosion trend, i.e., the more positive the value tends, the better the tested surface is protected from further corrosive attacks.

### 3.4. Electrochemical Impedance Spectroscopy

**Figure 7:**
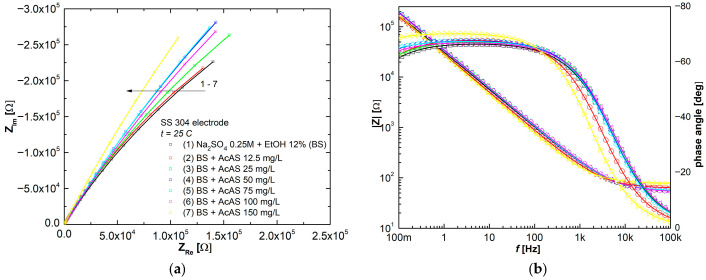
(**a**) Nyquist and (**b**) Bode plots of stainless steel 304 in Na_2_SO_4_ 0.25 mol L^−1^ with 12% EtOH (BS) test solutions, in the absence and presence of different concentrations of AcAS at 25 °C. (Open symbols show experimental values, and continuous lines were obtained by fitting.)

**Figure 8:**
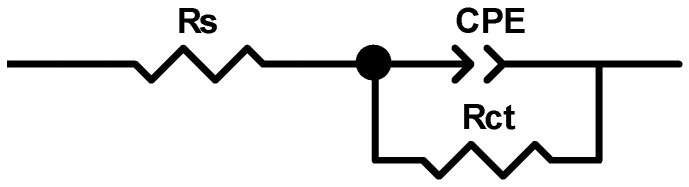
Electrical equivalent circuit (EEC) for modeling SS304 corrosion processes in Na_2_SO_4_ 0.25 mol L^−1^ with 12% EtOH (BS) test solutions.

Figure 7a,b present EIS spectra depicted as Nyquist and Bode plots illustrating SS 304 corrosion in Na_2_SO_4_ 0.25 mol L^−1^ with 12% EtOH (BS) test solutions in the absence and presence of the different AcAS concentrations used in the experimental studies, at the same temperature of 25 °C. The continuous lines were generated by fitting, using a Randles circuit from Figure 8, while open symbols indicate experimental data. The color code used in the Bode plots is the same as that shown in the Nyquist plot legend.

The equivalent electrical circuit (EEC) shown in Figure 8 comprises a solution resistance *R*_s_ in series with a parallel connection of a constant phase element (*CPE*), which accounts for the double layer capacity and a series connection between a charge transfer resistance *R*_ct_ and the magnetic coil (inductor) element. This is a typical electrical circuit for the adsorption process of inhibitors on metal surfaces, where chemical species, ions or other molecules are physically adsorbed at the metallic interface of the electrochemical double layer with a given electrical charge motion [27,33].

The resistance *R*_s_ component represents the uncompensated solution resistance. In the EEC used for simulating the SS304 corrosion process, the ideal capacitor characterized by double-layer capacity (*C*_dl_) is replaced by a constant phase element (*CPE*) because it more precisely represents the real electrochemical system behavior, particularly of depressed loops in EIS spectra.

The impedance of *CPE* is described by Equation (2).

(2) $$Z_{CPE}=1/T{\left(j\omega \right)}^{n}$$

where *T* is a parameter proportional to double-layer capacity, and n is an exponent which describes the *CPE* angle with values between 0 and 1 [34,35].

Double-layer capacity values (*C*_dl_) were calculated using Equation (3).

(3) $$C_{dl}=T^{1/n}{\left(\frac{1}{R_{s}}-\frac{1}{R_{ct}}\right)}^{\frac{n-1}{n}}$$

The inhibition efficiency values of the AcAS compound were determined using the charge transfer resistance (*R*_ct_) values from Table 3 for SS304, following Equation (4), where *R*^0^_*ct*_ and *R*^*i*^_*ct*_ are the charge transfer resistances in the test solution with and without the different AcAS concentrations, respectively.

(4) $$IE\;\left(\%\right)=\frac{\left(\frac{1}{R_{ct}^{0}}\right)-\left(\frac{1}{R_{ct}^{i}}\right)\;}{\left(\frac{1}{R_{ct}^{0}}\right)}\cdot 100$$

The diameter of the pseudo-semicircle shows a correlation with the *R*_ct_ value, which increases slightly as the concentration of AcAS increases. This indicates that charge transfer becomes progressively inhibited in the presence of AcAS. This observation is further supported by the shift in the characteristic frequency of the charge transfer process towards lower values with increasing AcAS concentration. As illustrated in the Bode plots (Figure 7b), the magnitude of the low-frequency impedance increases proportionally with the concentration of AcAS in the blank solution. Likewise, the phase angle grows in the presence of AcAS, while the frequency at which the maximum occurs moves to lower values, confirming the inhibitory efficiency of AcAS.

In the process modeled by the EEC presented in Figure 8, the *R*_ct_ parameter exhibits a direct relationship with the corrosion resistance of SS304 specimens immersed in 0.25 mol L^−1^ Na_2_SO_4_ containing 12%EtOH (BS). The fitting results summarized in Table 3 clearly demonstrate that *R*_ct_ increases progressively with higher AcAS concentrations in the test solutions, thereby substantiating the conclusions drawn from linear polarization measurements. Furthermore, the low chi-square values, in the order of 10^−3^, confirm excellent agreement between the experimental EIS data and the values predicted by the EEC model.

### 3.5. Molecular Modeling

**Figure 9:**
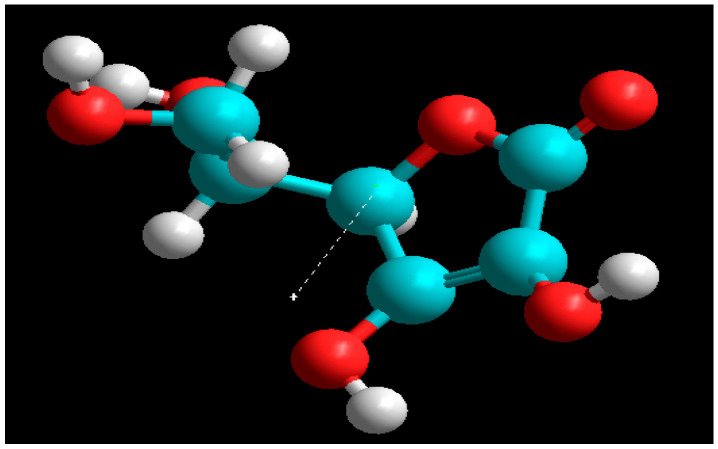
Optimized structure of the AcAS molecule.

**Figure 10:**
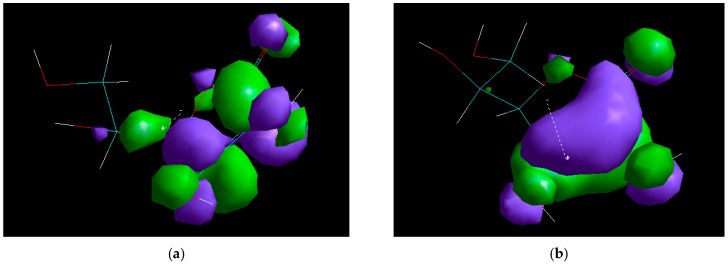
Frontier orbitals of the AcAS molecule: (**a**) H.O.M.O., (**b**) L.U.M.O.

The molecular modeling technique was used as a complement to the studies derived from the electrochemical techniques used. The study was carried out using the Hyperchem 8.0 software package, using the DFT density function method with the B3LYP functional and the 6-31G* base set on the AcAS molecule, which was modeled under vacuum and water environment conditions. Figure 9 shows the AcAS molecule, with the orientation of the dipole moment shown by the direction of the dotted arrow in the figure, from − to +. The representation of the atoms in the structure of AcAS was made according to specific color codes: carbon atoms were shown in turquoise, hydrogen atoms in white, and oxygen atoms in red. Subsequent to the optimization, the energies of the *E*_HOMO_ (Figure 10a) and *E*_LUMO_ (Figure 10b) molecular orbitals were acquired, according to the molecular descriptors presented in Table 4.

The distribution of the Frontier orbitals indicates that the H.O.M.O. is primarily concentrated on the enediol fragment of AcAS and the oxygen heteroatoms, signifying that these regions are responsible for the electron donor properties and adsorption onto the metal surface. The L.U.M.O. is predominantly distributed on the lactonic cycle and carbonyl group, which results in the electrophilic character of the molecule and the acceptance of electron density from the metal surface [36].

The data in Table 4 demonstrated that the *E*_HOMO_ energy (−8.82 eV) indicates a weak capacity of the AcAS molecule to donate electrons, while *E*_LUMO_ (−1.45 eV) suggests a moderate electron acceptance capacity [37]. The ΔE energy gap of 7.37 eV is considerable and indicates high chemical stability and low reactivity, which characterizes AcAS as a passive inhibitor that adsorbs to the metal surface, without aggressive redox processes [38]. The dipole moment of the molecule is moderate (3.14 Debye), suggesting an acceptable polarization and the possibility of electrostatic interactions with the active centers of the metal surface [39]. Electronegativity (χ = 3.69 eV) and low chemical hardness (σ = 0.195 eV^−1^) indicate the integrity of the molecule and resistance to spontaneous electronic reactions [35]. The QSAR properties, namely the molecular volume V = 485.89 Å^3^ and surface molecular area S = 324.8 Å^2^, indicate a reasonable covering capacity of the metal surface. The V/S ratio of around 1.5 confirms that AcAS may establish a homogenous adsorbed layer, possibly obstructing the corrosive species in the environment. Therefore, the data obtained through the molecular modeling technique confirm that AcAS has favorable structural characteristics in the sense of being used as a corrosion inhibitor; specifically, it is stable, chemically reactive and capable of forming effective protective films, due to its moderate size and polarity.

### 3.6. Adsorption Isotherms

**Figure 11:**
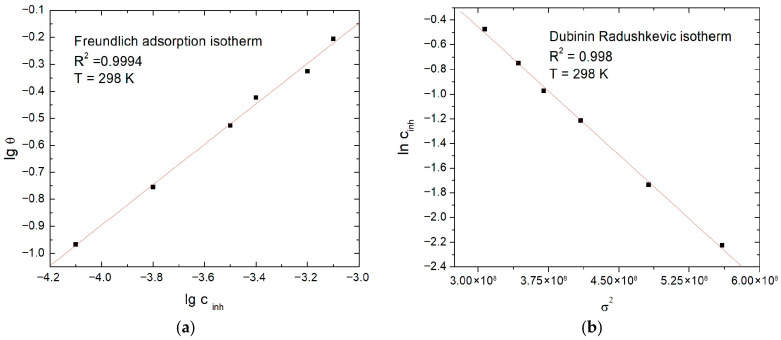
Adsorption isotherms: (**a**) Freundlich and (**b**) Dubinin–Radushkevic isotherms.

In order to evaluate the nature and intensity of the interactions between the AcAS molecules and the metallic surface of SS304 in BS, various models of adsorption isotherms were plotted, including: Langmuir, Freundlich, Temkin, Frumkin, Flory–Huggins (F-H), El Awady, Dubinin–Radushkevich (D-R) and Volmer, by using the degrees of coverage θ obtained by calculations based on the corrosion current densities obtained by linear sweep voltammetry measurements at AcAS concentrations of 12.5, 25, 50, 75, 100 and 150 mg/L, where the corrosion current is a gross determination based on the balance between the anodic and the cathodic reactions [40]. The plotted isotherms describe potential physicochemical scenarios of adsorption, from the formation of a single ideal layer on homogeneous surfaces to adsorption on heterogeneous surfaces, with lateral interactions or the formation of layers consisting of several molecules [41]. Based on the R^2^ calculation from the plotted isotherms (Table 5), the Freundlich and Dubinin–Radushkevic isotherms were found to describe the adsorption behavior of AcAS on SS304 (Figure 11).

The Freundlich isotherm describes the adsorption on non-equivalent energy sites, an adsorption characteristic of heterogeneous surfaces. Its linear form, describing the relationship between the coverage degree and the concentration of the inhibitor, is described by Equation (5) [42].

(5) $$\log\theta =\log K_{F}+\frac{1}{n}\log c_{inh}$$

where *K*_F_ represents the Freundlich constant, and 1/*n* is the heterogeneity of the surface. The experimental data are in correlation with the linear representation log *θ* = f(log *c*_i__nh_). From the value of n = 1.42 (>1), a heterogeneous favorable adsorption is suggested, and the *K*_ads_ constant is 91.66 L mol^−1^. Based on *K*_ads_, the standard free energy $∆G_{ads}^{o}$ was calculated with Equation (6) [43].

(6) $$∆G_{ads}^{o}=-RTln(55.55K_{ads})$$

where R = 8.314 J mol^−1^ K^−1^, T—thermodynamic temperature [K], and *K*_ads_—adsorption constant [L mol^−1^].

The parameters obtained from the fitting of the Freundlich isotherm are presented in Table 6.

The value $∆G_{ads}^{o}$ equal to −21.1 kJ mol^−1^ is situated at the lower limit of the characteristic range of the mixed adsorption process, indicating that the physical dominance is in agreement with the moderately strong, but spontaneous nature of the AcAS-SS304 interactions [44].

The Dubinin–Radushkevich (D-R) isotherm also describes adsorption on heterogeneous surfaces, characterized by a Gaussian distribution of adsorption energies, usually associated with physisorption. The linear form is described by Equation (7) [45].

(7) $$\ln\theta =\ln\theta_{max}+\alpha_{D}\cdot \sigma^{2}$$

where $\alpha_{D}$ is the D-R constant, and σ is the Polanyi potential that depends on the concentration of the inhibitor. The parameters obtained at 298 K are shown in Table 7.

The adsorption energy resulting from the D-R model falls within the specific domain of physiosorption, supporting a predominantly physical adsorption mechanism at the AcAS–metal interface. The high correlation coefficient of 0.9984 supports the suitability of the model for describing experimental data [45].

From the comparative analysis of the R^2^ correlation coefficients for all the tested models, it appears that the Freundlich and Dubinin–Radushkevich isotherms provide the best statistical description of the experimental data, followed by the El-Awady model and later by Langmuir. The unit proximity of R^2^ for Langmuir (0.9509) indicates a significant contribution of a single-layer adsorption process, but the superior correlation of the Freundlich and D-R models suggests that AcAS interacts with a far-from-ideal surface, with unequivalent adsorption sites and a wide distribution of binding energies.

## 4. Conclusions

The electrochemical study reveals that AcAS is an electroactive compound with an inhibitory effect relevant to steels utilized in the food industry, such as SS304. Cyclic voltammetry on the Pt electrode has the role of highlighting the anodic processes associated with the oxidation of AcAS as a function of concentration. In the cathodic field, it demonstrates adsorption effects at higher concentrations; respectively, the displacement of the hydrogen release potentials tends towards more negative potential values as a result of the covering of the active surface.

The potentiodynamic polarization determinations confirm the decrease in the *v*_corr_ on SS304 in the working environment in the presence of AcAS, concomitant with the notable shift in *E*_corr_ towards more positive values. From the simultaneous decrease in the anodic and cathodic Tafel slopes, a mixed inhibition mechanism results, with an important anodic passivation component. Studies conducted at temperatures in the range of 298–328 K confirm the protective effect of AcAS. Although corrosion processes are thermally activated, higher concentrations of AcAS maintain inhibition more efficiently by forming a stable adsorbed layer through possible weak interactions or covalently coordinative bonds. From the results of the chronoamperometry or chronopotentiometry determinations, the consolidation of the protective film is supported by decreasing the anode currents and moving the *E*_OCP_ towards more positive values and reaching a quasi-stationary regime.

From the electrochemical impedance spectroscopy (EIS) experiments, the inhibitory effect of AcAS, demonstrated in electrochemical experiments, is confirmed. In the Nyquist spectra, an increase in the diameter of the semicircle is observed, corresponding to the increase in the resistance to charge transfer *R*_c__t_, therefore, indicating the progressive inhibition of corrosion processes. In Bode diagrams, the increase in impedance at low frequencies and phase angle is observed, with the shift in frequency towards lower values.

Molecular modeling supports the proposed mechanism, suggesting that, based on the distribution of the H.O.M.O. and L.U.M.O. orbitals, AcAS has active centers on oxygen-rich groups capable of interacting with the metal surface. The energy gap of 7.37 eV indicates the high chemical stability specific to a passive inhibitor, which forms an adsorbed film without aggressive redox processes. The QSAR parameters (V, S, and V/S) demonstrate optimal dimensions for the establishment of a dense layer that can obstruct the ingress of corrosive agents.

From the study of adsorption isotherms, we concluded that the adsorption at the SS304 level of AcAS is spontaneous and mostly heterogeneous. The Freundlich and Dubinin–Radushkevich models best describe the dataset, and the parameters of the Freundlich isotherm indicate favorable adsorption on heterogeneous surfaces and the value of the standard Gibbs free energy, $\Delta G_{ads}^{o}$ = −21.1 kJ mol^−1^, corresponds to a physisorption process with a combined physicochemical component. The adsorption energy in the Dubinin–Radushkevich model confirms the existence of physical interactions (Van der Waals forces and hydrogen bonds) at the metal–solution interface with the inhibitor.

From an application perspective, SS304 is a food-grade stainless steel used in the wine industry for constructing components involved in the technological flow (fermentation and storage tanks, pipes, and heat exchangers), whereas AcAS is added as an antioxidant in winemaking and wine preservation. The findings of this study indicate that under similar conditions, the presence of AcAS in the environment can contribute not only to the oxidative stability of the wine but also to the passivation of the metal surface, reducing the corrosion rate and, implicitly, the risk of release of metal ions into the product. In this sense, AcAS plays a dual role: as a dietary antioxidant and corrosion inhibitor, a crucial aspect for the design and use of equipment in the wine industry in conditions of durability and safety.

## Figures and Tables

**Table 1:** Elemental composition of SS304 steel.

Cr (wt%)	Ni (wt%)	C (wt%)	Mn (wt%)	Si (wt%)	N (wt%)	P (wt%)	S (wt%)
18–20	8–10.5	0.07	2	1	0.11	0.045	0.03

**Table 2:** Kinetic characteristics of Tafel slopes and their temperature dependence.

**Electrolyte**	***E*_corr_ [mV]**	***i*_corr_** **[µA cm^−2^]**	***β*_a_**	***β*_c_**	***v*_corr_** **[mm y^−1^]**	***IE* [%]**	**θ**
BS	631	14.8·10^−3^	69.8	17.7	0.158·10^−3^	-	-
BS + 12.5 mg/L AcAS	666.7	13.2·10^−3^	63.3	16.9	0.134·10^−3^	10.8	0.108
BS + 25 mg/L AcAS	693.5	12.2·10^−3^	59.9	15.7	0.124·10^−3^	17.6	0.176
BS + 50 mg/L AcAS	731.9	10.4·10^−3^	54.3	14.8	0.103·10^−3^	29.7	0.297
BS + 75 mg/L AcAS	759.4	9.2·10^−3^	49.3	14.2	82.92·10^−6^	37.8	0.378
BS + 100 mg/L AcAS	798.7	7.8·10^−3^	43.6	13.4	61.74·10^−6^	47.3	0.473
BS + 150 mg/L AcAS	926.3	5.6·10^−3^	40.6	12.4	43.01·10^−6^	62.2	0.622
**Electrolyte**	**t [°C]**	***E*_corr_ [mV]**	***i*_corr_ [µA cm^−2^]**	***β*_a_**	***β*_c_**	***v*_corr_** **[mm y^−1^]**	
BS	25	631	14.8·10^−3^	69.8	17.7	0.158·10^−3^	
	35	417.7	16.4·10^−3^	75.5	19.9	0.166·10^−3^	
	45	385.7	20·10^−3^	89.1	23.9	0.207·10^−3^	
	55	365.6	26·10^−3^	90.1	42.6	0.269·10^−3^	
BS + 75 mg L^−1^ AcAS	25	759.4	9.2·10^−3^	49.3	14.2	0.082·10^−3^	
	35	673.7	10.2·10^−3^	52.4	18.4	0.103·10^−3^	
	45	620.8	14·10^−3^	67.8	20.6	0.145·10^−3^	
	55	575.2	18.4·10^−3^	97.1	24.5	0.186·10^−3^	
BS + 150 mg L^−1^ AcAS	25	926.3	5.6·10^−3^	40.6	12.4	0.043·10^−3^	
	35	881	8·10^−3^	41.5	15.5	0.082·10^−3^	
	45	842.9	12·10^−3^	54.1	20.4	0.124·10^−3^	
	55	774.1	14·10^−3^	68.3	28.5	0.145·10^−3^	

**Table 3:** Impedance parameters for SS304 corrosion at 25 °C in Na_2_SO_4_ 0.25 mol L^−1^ with 12% EtOH (BS) test solutions in the absence and presence of varying concentrations of AcAS calculated by fitting the experimental data.

**AcAS Conc.** **(mg L^−1^)**	***R*_s_** **(Ω)**	***CPE-T*** **(µF cm^−2^ sn^−1^)**	***n***	***R*_ct_** **(Ω cm^2^)**	**Chi^2^·10^3^**
BS	61.9 (1.32%)	8.56 (1.23%)	0.761 (0.30%)	1.44·10^6^ (1.51%)	0.54
12.5	52.4 (1.82%)	8.24 (1.62%)	0.755(0.38%)	1.61·10^6^ (2.08%)	0.86
25	52.2 (2.09%)	7.92 (1.77%)	0.753 (0.42%)	1.76·10^6^ (2.46%)	1.04
50	53.8 (1.98%)	7.75 (1.69%)	0.750 (0.40%)	2.07·10^6^ (1.05%)	0.93
75	54.2 (1.86%)	7.59 (1.59%)	0.744 (0.38%)	2.38·10^6^ (2.75%)	0.89
100	51.9 (2.15%)	7.46 (1.73%)	0.739 (0.41%)	2.76·10^6^ (2.23%)	1.10
150	66.24 (0.87%)	7.25 (0.98%)	0.735 (0.19%)	3.92·10^6^ (2.97%)	0.33
**AcAS Conc.** **(mg L^−1^)**		***C*_dl_·10^5^** **(µF cm^−2^)**		***E* (%)**	**θ**
BS		1.88		–	–
12.5		1.89		10.60	0.11
25		1.91		18.41	0.18
50		1.95		30.60	0.31
75		2.05		39.57	0.40
100		2.17		47.89	0.48
150		2.42		63.32	0.63

**Table 4:** Values of the molecular descriptors related to the AcAS molecule.

Descriptor	Value
E_HOMO_ (eV)	−8.82
E_LUMO_ (eV)	−1.45
ΔE (eV)	7.37
Dipole moment *µ* (Debye)	3.14
Absolute electronegativity (eV)	3.68
Chemical hardness η (eV)	5.14
Softness (eV^−1^)	0.194
Molecule volume V (Å^3^)	485.89
Molecule surface (Å^2^)	324.8
Ratio V/S (Å)	1.495

**Table 5:** The R^2^ values for the models plotted.

Adsorption Model	Freundlich	D-R	El-Awady	Langmuir	Frumkin	Temkin	F-H	Hill de Boer
R^2^	0.9994	0.9984	0.9869	0.9509	0.9432	0.9432	0.8815	0.8551

**Table 6:** The parameters of the Freundlich isotherm.

a (log *K*_F_)	b	R^2^	n	*K*_ads_ [L mol^−1^]	$∆G_{ads}^{o}$ [kJ mol^−1^]
1.9622	0.7054	0.9994	1.4176	91.66	−21.147

**Table 7:** Parameters of D-R isotherm.

T [K]	a	b	$\alpha_{D}$	*E*_ads_ [J mol^−1^]	R^2^
298	1.5518	−7·10^−9^	7·10^−9^	8451.54	0.9984

## Data Availability

The original contributions presented in this study are included in the article. Further inquiries can be directed to the corresponding author.
